# Dishevelled family proteins (DVL1-3) expression in IUGR placentas

**DOI:** 10.17305/bjbms.2020.5422

**Published:** 2021-08

**Authors:** Ida Marija Sola, Alan Serman, Valentina Karin-Kujundzic, Frane Paic, Anita Skrtic, Paula Slatina, Luka Kakarigi, Semir Vranic, Ljiljana Serman

**Affiliations:** 1 Department of Obstetrics and Gynecology, University Hospital “Sestre Milosrdnice,” Zagreb, Croatia; 2 Department of Gynecology and Obstetrics, School of Medicine, University of Zagreb, Zagreb, Croatia; 3 Clinic of Obstetrics and Gynecology, Clinical Hospital “Sveti Duh,” Zagreb, Croatia; 4 Centre of Excellence in Reproductive and Regenerative Medicine, University of Zagreb School of Medicine, Zagreb, Croatia; 5 Department of Biology, School of Medicine, University of Zagreb, Zagreb, Croatia; 6 Department of Pathology, School of Medicine, University of Zagreb, Zagreb, Croatia; 7 Department of Pathology, University Hospital “Merkur,” Zagreb, Croatia; 8 College of Medicine, QU Health, Qatar University, Doha, Qatar; 9 Biomedical and Pharmaceutical Research Unit, QU Health, Qatar University, Doha, Qatar

**Keywords:** Intrauterine growth restriction, placenta, Dishevelled family proteins, DVL1, DVL2, DVL3

## Abstract

Dishevelled family proteins (DVL1, DVL2, and DVL3) are cytoplasmic proteins that are involved in canonical and non-canonical Wnt signaling pathway during embryonic development. The role of DVL proteins in the placental tissue remains mostly unknown. In the current study, we explored the role of Dishevelled proteins in naturally invasive tissue, trophoblast. Formalin-fixed paraffin-embedded samples of 15 term placentas from physiologic term pregnancies and 15 term placentas from pregnancies complicated with intrauterine growth restrictions (IUGR) were used for the study. Expression levels of mRNA for *DVL1*, *DVL2*, and *DVL3* in placentas were analyzed by quantitative real-time PCR (qRT-PCR). DVL1, DVL2, and DVL3 protein expression were semi-quantitatively analyzed using immunohistochemistry. The expression of DVL3 protein was significantly higher in trophoblasts and endothelial cells in placental villi from IUGR pregnancies compared with the control group of term placentas, while DVL2 protein expression was significantly higher in trophoblasts in placental villi from IUGR pregnancies compared with normal term placentas. The observed differences at protein levels between normal and IUGR placentas were not confirmed at the mRNA levels of *DVL* genes. Our data indicate the active involvement of DVL proteins in IUGR-related placentas. No significant changes were observed in *DVL* mRNA levels between the two groups of placentas. Further studies are required to explore the clinical relevance of these observations.

## INTRODUCTION

The invasion into the endometrium and remodeling of the mother’s spiral arteries are essential steps for the proper functions of the placenta during pregnancy [1]. Trophoblast invasion is a spatially and temporally precisely regulated process so that it does not end up too shallow, as is the case in preeclampsia and in intrauterine growth restriction (IUGR), or too deep, as it occurs in placenta percreta and placenta accreta.

IUGR is defined as a condition in which the fetus cannot achieve his full, genetically determined growth potential [2], thus causing long- and short-term morbidity and mortality [3]. De Jesus *et al*. found an almost 4-fold higher risk of neurodevelopmental impairment or neonatal death and 2.6-fold increased risk of cerebral palsy in IUGR neonates born before 27 weeks’ gestation compared with age-matched non-small for gestational age neonates [4].

Wnt signaling pathway is, along with Hippo, Hedgehog, Notch, and TGFß pathways, one of the evolutionarily preserved pathways that are essential for embryonic development, control of early axis formation and organogenesis [5,6] by enabling cell differentiation, proliferation, migration, polarity, and survival [7].

Wnt intracellular signaling transmission and interpretation are mediated by a family of cytoplasmic proteins called Dishevelled (DVL), human homologs of the Drosophila Dishevelled gene (dsh) – DVL 1, DVL 2, and DVL 3, through secreted frizzled-related proteins (SFRP) at the cell membrane [7,8].

We have previously shown increased activation of the SFRP family members, inhibitors of the Wnt signaling pathway, in pathological placentas from pregnancies with IUGR [9]. In addition, it has been found that mice with hypermethylated *Wnt2* gene promoter have a lower birth weight (they are small for gestational age, SGA) [10]. In contrast, another gene, *Wnt7b*, turns out to be necessary for the fusion of chorion and allantois in mice [11].

In the current study, we explored the status of DVL1-3 in a naturally invasive placental tissue using quantitative real-time PCR (qRT-PCR) and immunohistochemistry. Our goal was to understand the patterns of DVL protein expression in IUGR placentas and compare it with placentas from physiological pregnancies.

## MATERIALS AND METHODS

### Materials

The samples used in the study were a part of a collection of placental tissue samples belonging to the University of Zagreb School of Medicine and had been collected in collaboration with the University Hospital “Merkur” Zagreb, both of which are parts of the Scientific Center of Excellence for Reproductive and Regenerative Medicine. This study was approved by the Ethical Committees of the School of Medicine, University of Zagreb and the University Hospital “Merkur” and was performed according to ethical standards of the Declaration of Helsinki.

In the examination of placentation, a control group consisted of formalin-fixed paraffin-embedded (FFPE) tissue samples of 15 placentas, obtained from complication-free pregnancies, physiological singleton, and delivered at term (between 38 and 42 weeks of gestation) of a newborn with normal body weight (between 10^th^ and 90^th^ percentile for gestational age, newborn sex, and mother’s parity). The experimental group consisted of 15 term placentas from pathological pregnancies with fetal growth restriction (FRG, IUGR) observed on serial ultrasound (at least twice), with the assessment of the bodyweight below 10^th^ percentile for the duration of pregnancy, fetal sex, mother’s parity, and confirmed at birth by measuring newborn body weight. Only pathological pregnancies with IUGR were included, and exclusion criteria for both pathological pregnancies and controls were as follows: Multiple pregnancies, tobacco and drug use, intrauterine viral infections (TORCH and Parvovirus B19), chorioamnionitis, hypertension, preeclampsia, fetal malformations, and genetic abnormalities as well as autoimmune diseases or eating disorders of the mother.

The board-certified pathologist (A.S.) examined each placenta and rendered the diagnosis. A disc-shaped tissue sample, comprising an entire thickness of the placenta from fetal to maternal side, about 5 cm from the umbilical cord, was taken from each placenta.

### Immunohistochemistry (IHC) for DVL1-3 proteins

Deparaffinized placental tissue sections (4 μm thickness) were mounted on glass slides (Agilent Dako, Carpinteria, CA, United States). The slides were conjugated with primary antibodies for 30 min at room temperature, followed by antibody detection using Dako REAL Envision detection system (Agilent Dako, Carpinteria, CA, United States). Citric buffer pH = 6 (Agilent Dako, Carpinteria, CA, United States) was used for antigen retrieval. Primary antibodies (all from Santa Cruz Biotechnology, Dallas, TX, USA) were mouse monoclonal anti-human DVL1 (dilution 1:100, Cat No. sc-8025), rabbit polyclonal anti-human DVL2 (dilution 1:200, Cat No. sc-13974), and mouse monoclonal anti-human DVL3 (dilution 1:200, Cat No. sc-271295). Negative controls were processed for all assays and were done in the same manner except incubation step with primary antibody, which was omitted.

### Quantitative analysis of DVL1-3 protein expression

The expression of DVL1, DVL2, and DVL3 in placentas was independently assessed by two board-certified pathologists (A.S., S.V.). The tissue compartments (trophoblasts, stromal cells, and endothelial cells) were scored as follows: 0 if no staining was observed; 1 if <10% cells were stained; 2 if 10-50% cells were stained; and 3 if >50% cells were stained [12]. All the discordant result was resolved at the double-headed microscope evaluation.

### RNA extraction, reverse transcription, and qRT-PCR

Total RNA was isolated from 5 × 5 μm sections of IUGR (n = 15) and control placental (n= 15) FFPE tissue blocks. Shortly, all samples were deparaffinized using 1.0 mL xylene (Invitrogen, UK), followed by incubation for 3 min at 50°C and centrifugation for 5 minutes at maximum speed. The supernatant was then discarded, and the pellet was washed twice with 1.0 mL absolute ethanol. The samples were incubated with 300 μL of protease K digestion buffer (20 mM Tris-HCl [pH 8.0]; 1 mM CaC_l2_; 0.5% sodium dodecyl sulfate and 500 μg/ml protease K) for 3h at 55°C. Subsequently, RNA was isolated with TRIzol reagent (Invitrogen Life Technologies, Carlsbad, CA, USA) according to the manufacturer’s instructions. RNA purity and concentration were evaluated by NanoDrop 2000 spectrophotometer (Thermo Fisher Scientific, United States). Two micrograms of total RNA from each sample were reverse transcribed using the high capacity cDNA Reverse Transcription Kit (Applied Biosystems, Thermo Fisher Scientific, United States), following the manufacturer’s protocol. *DVL1*, *DVL2*, and *DVL3* gene expression were quantified using the CFX-96 real-time PCR detection system using a C100 thermal cycler (Bio-Rad Laboratories, United States). All qPCR reactions were performed in triplicates in the presence of the TB Green™ Premix Ex Taq™ II (Tli RNaseH Plus PCR master mix, Takara Biotechnology Co., Ltd.) under the following thermocycler conditions: Stage 1: 95°C for 30 s, one cycle; stage 2: 95°C for 5 s and 60°C for 30 s, 40 cycles. The CFX96 manager software was used to generate the cycle threshold (Ct) values, and the data were analyzed by the 2-ΔΔCT method. The relative expression of targeted *DVL1-3* genes was normalized against the β-actin (*ACTB*) gene that served as an endogenous control [12]. The specificity of qPCR amplification was confirmed using melt curve analysis. The sequence of used oligonucleotide primers were as follows: DVL1 FW: 5’-CCCCTCCTTCCACCCAAATG-3’, RW:5’-GTGACTGACCATGGACTCCG-3’ (Accession:NM_001330311.2);DVL2FW:5’-TGAGCAACGATGACGCTGTG-3’,RW:5’-GCAGGGTCAATTGGCTGGA-3’ (Accession: NM_004422.3); DVL3 FW: 5’-ACAATGCCAAGCTACCATGCTTC-3’, RW:5’-AGCTCCGATGGGTTATCAGCAC-3’ (AccessionNM_004423.4); ACTB FW:5’- GGGCATGGGTCAGAAGGATT-3’, RW: 5’-AGTTGGTGACGATGCCGTG-3’ (Accession: NM_001101) [12].

### Statistical analysis

The Kolmogorov–Smirnov test and Shapiro–Wilk W-test assessed the distribution of the data. Student’s *t*-test analyzed the clinical data of normal and IUGR pregnancies. A Mann–Whitney test was used for comparison among placental expression of DVL1, DVL2, and DVL3 proteins, with significant differences accepted at a probability value of p < 0.05. The data were analyzed using the GraphPad Prism 5.01 program (GraphPad Software, Inc., San Diego, CA, USA) and IBM SPSS Statistics, Version 21.

## RESULTS

### Clinical data – normal and IUGR pregnancies

Thirty term placentas were studied, of which 15 were from pregnancies with IUGR and 15, serving as controls, were from healthy pregnancies. The following clinical variables were analyzed: Age, blood pressure, body weight and height of pregnant women, body weight gain, and body mass index before gestation and at time of delivery, fetal body weight and height, placental weight, and fetal/placental weight ratio (Table 1). As expected, a statistically smaller fetal weight and height and placental weight were found in newborns from IUGR pregnancies (p < 0.0001). The mean age of pregnant women with IUGR was 32 years compared with healthy controls (28 years; p = 0.073).

**TABLE 1:**
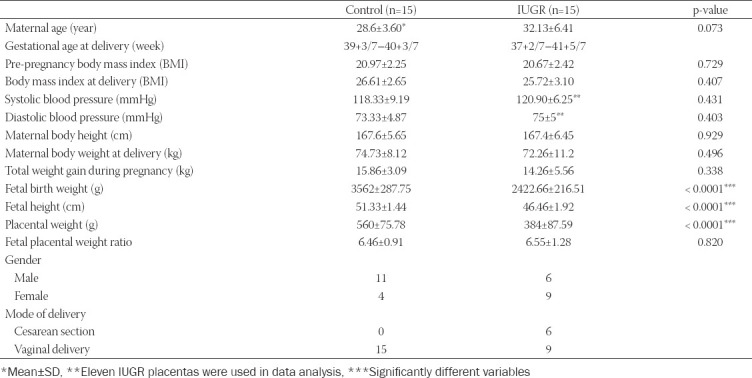
Clinical parameters of mothers and children from normal (control) and pathologic (intrauterine growth restriction – IUGR) term pregnancies

### Expression of Dishevelled family proteins in IUGR placentas

**FIGURE 1:**
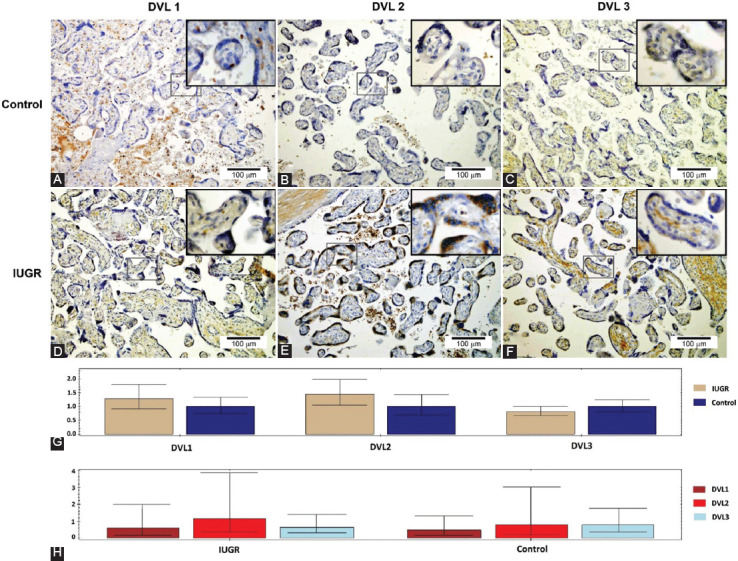
DVL1, DVL2, and DVL3 protein expression in human term placentas from normal pregnancies – control (A-C) and human term placentas from intrauterine growth restriction pregnancies (D-F). Relative versus normalized expression values of DVL1, DVL2, and DVL3 mRNA in IUGR versus control placental tissue (G) and their expression inside the individual tissue sample group (H).

**FIGURE 2:**
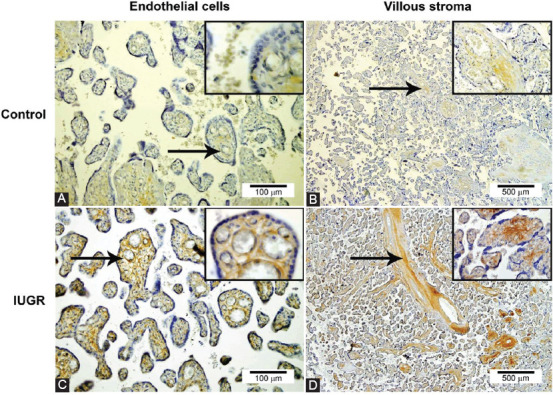
DVL3 protein expression in endothelial cells (arrows) in placental villi from normal (control) and intrauterine growth restriction pregnancies (A and C), and DVL3 protein expression in stem (arrows) and terminal villous stroma in the normal (control) and intrauterine growth restriction placentas (B and D).

Expression of all three Dishevelled proteins was detected in the cytoplasm of both trophoblasts and stromal cells of the placental villi (Figure 1A-F). In IUGR placentas, DVL2 and DVL3 were expressed in >10% of trophoblast cells in 100% and 66.7% of samples, respectively, while in normal placentas, DVL2 and DVL3 were expressed in <10% of epithelial cells in 26.6% and 66.7% of samples, respectively (Table 2). Expression of DVL2 and DVL3 was significantly lower in trophoblasts in placental villi from uncomplicated pregnancies (Figure 1B and C) than in trophoblasts in placental villi from IUGR pregnancies (p = 0.016 and p = 0.030, respectively) (Figure 1E and F). DVL3 protein expression was significantly higher in endothelial cells in placental villi from IUGR pregnancies compared with endothelial cells in placental villi from uncomplicated pregnancies (p = 0.021) (Figure 2A and C). Intense staining of DVL3 protein was present in stem villous stroma in the IUGR placentas. Simultaneously, there was a weaker staining in the terminal villous stroma in the IUGR placentas and very weak staining in stem and terminal villous stroma in the normal placentas (Figure 2B and D). In term placentas with IUGR, the pattern of DVL proteins expression was DVL3< DVL1< DVL2. Thus, there was a statistically significant difference in expression among the IUGR group between DVL1 and DVL2 (p = 0.004) and between DVL2 and DVL3 (p = 0.002), whereas between DVL1 and DVL3, such difference was not statistically significant (p = 0.863) (Figure 1D-F). There was no statistically significant difference in DVL proteins’ expression among normal term placentas (Figure 1A-C).

**TABLE 2:**
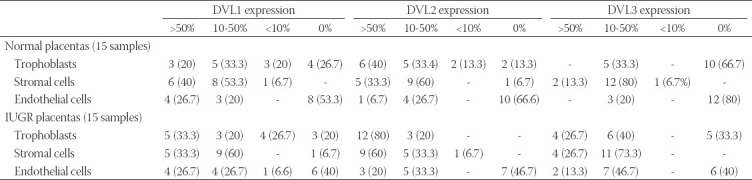
Expression of DVL1, DVL2, and DVL3 proteins in term placentas from normal pregnancies and term placentas from IUGR pregnancies

### Expression levels of mRNA for DVL1, DVL2, and DVL3 in IUGR placentas

The qRT-PCR analysis results showed that all three human *DVL* gene homologs were transcriptionally active in both IUGR and healthy control tissue samples (Figure 1G and H). The relative mRNA expression levels of DVL1 and DVL2 were 1.28 fold and 1.44 fold higher in the IUGR than the control tissue samples. In contrast, DVL3 transcripts were reduced in IUGR samples compared with the control placental tissues (Figure 1G). However, none of the observed differences in mRNA expression levels was statistically significant. Regarding the mRNA expression levels of *DVL* homologs, in IUGR tissue samples analyzed as a separate group, the *DVL2* gene showed the highest transcriptional activity. In contrast, the expression levels of *DVL2* and *DVL3* in the control tissue group were almost identical (Figure 1H).

## DISCUSSION

This study aimed to determine the expression of Dishevelled proteins and their mRNA in healthy placentas and placentas with intrauterine growth restriction (IUGR). Since the placenta needs to continually adapt during its development to the demands placed on it by the growing embryo/fetus, the cells that contribute most to this adaptability, the trophoblast cells, must be under constant control a series of molecular mechanisms [13]. One such mechanism is the Wnt signaling pathway, whose central mediators are the DVL proteins.

One of the critical events during placentation is the epithelial-mesenchymal transition (EMT), which allows interstitial and endovascular invasion of the trophoblast cells. The reverse process, the mesenchymal-epithelial transition, contributes to stopping the invasion [14]. The timely progress of these processes is a prerequisite for successful placentation and, consequently, a healthy child’s birth. It has been previously demonstrated that unsuccessful trophoblast invasion occurs in placentas associated with fetal IUGR and hypertensive disorders [15,16].

Wnt signaling promotes cell motility and invasion [17]. SFRP proteins that represent the Wnt pathway’s essential inhibitors are more pronounced in IUGR placentas than in normal pregnancies, suggesting that the placental invasion regulation is one of the crucial factors during pregnancy [18].

Based on the current understanding, we hypothesized that in tissues with less prominent trophoblast invasiveness, that is, those resulting from a defective invasion of the decidua or placental bed, signal transmission through the Wnt/DVL signaling pathway will be impaired because of the diminished expression of DVL proteins. Surprisingly, we found a significant increase in the expression of DVL2 and DVL3 proteins in trophoblasts in placental villi from IUGR placentas, compared with the control term placentas and higher DVL3 protein expression in endothelial cells of placental villi from IUGR placentas, compared with the normal placentas. Intense staining of DVL3 protein was present in stem villous stroma in the IUGR placentas. These results are not in line with those where the expression of positive regulators of trophoblast invasion (e.g., SNAIL protein) is decreased [19] and those where the expression of negative regulators (e.g., ELF5) is increased [20]. We also observed the discrepancies between protein and mRNA levels of all three DVLs. The discrepancy in expression levels of mRNA for *DVL1, DVL2*, and *DVL3* genes and their protein expression may be due to the whole tissue sections analyzed by qRT-PCR. Simultaneously, immunohistochemical analysis enabled protein expression analysis in specified tissue compartments (trophoblasts vs. endothelial cells of placental villi) of IUGR and normal placental tissues.

Karimu and Burton previously showed that the pressure increased in the intervillous space reduced the fetal perfusion and increased the fetoplacental impedance by reducing fetoplacental capillaries width [21]. This mechanism has been proposed as a significant underlying cause of the IUGR. Several factors may affect the fetoplacental vasculature, including endocrine, metabolic, environmental, and oxygen factors [22]. Since a deficiency of oxygen in the tissue can be present in IUGR placentas, a lack of oxygen can stimulate capillaries’ growth to improve tissue oxygenation. Wnt signaling pathway induces vascular endothelial growth factor (VEGF) activation, one of the growth factors that drive angiogenesis [23]. This is in concordance with our results, which showed that increased expression of DVL3 protein in endothelial cells of placental villi from IUGR placentas might be associated with induced angiogenesis. During placental development, chorionic villi undergo developmental, transformative, regenerative, and reparative changes to establish a normal fetal blood flow. These changes can be observed in trophoblast cells and chorionic villi stroma because it is involved in the respective processes [24]. In IUGR placentas, these processes may be induced by unfavorable conditions (e.g., hypoxia), resulting in increased trophoblast cells and stroma in the chorionic villi. This is in line with our study that revealed a significant increase in DVL2 and DVL3 proteins’ expression in the trophoblast in placental villi from IUGR placentas and intense staining of DVL3 protein in stem villous stroma from IUGR placentas.

Steroids (estrogens and androgens) are actively involved in placental functions and pathologies [25,26]. It has been shown that DVL1 and DVL3 proteins could regulate placental aromatase expression and the aromatase gene transcription [27] that regulate human trophoblast differentiation [28]. Aromatase is an enzyme that converts androgens into estrogens, while estrogens promote cell proliferation and growth [24]. Besides, estrogens formed by placental aromatase may enhance placental angiogenesis [29]. These findings further support our view that overexpression of DVL3 protein in IUGR placentas might be associated with induced angiogenesis and increased activity of trophoblasts and stroma in the chorionic villi.

In conclusion, our study demonstrated the active and potentially complex roles that DVL proteins may play in IUGR-related pregnancies’ pathogenesis. Further studies should elucidate the clinical relevance of the observed DVL alterations.
